# Refractory ventricular arrhythmia in a patient with Lamin A/C (LMNA) cardiomyopathy successfully treated with thoracic bilateral stellate ganglionectomy

**DOI:** 10.1016/j.hrcr.2021.11.011

**Published:** 2021-11-24

**Authors:** Eze Okeagu, Ahad Abid, Brian C. Jensen, Thomas G. Caranasos, Faisal F. Syed

**Affiliations:** ∗ Division of Cardiology, University of North Carolina at Chapel Hill, Chapel Hill, North Carolina; † Department of Internal Medicine, University of North Carolina at Chapel Hill, Chapel Hill, North Carolina; ‡ Division of Cardiothoracic Surgery, University of North Carolina at Chapel Hill, Chapel Hill, North Carolina

**Keywords:** Cardiac sympathetic denervation, Lamin A/C (LMNA) cardiomyopathy, Stellate ganglionectomy, Sudden cardiac death, Ventricular fibrillation

## Introduction

Lamin A/C (*LMNA*) mutations cause familial dilated cardiomyopathy (DCM) with autosomal dominant inheritance and variable phenotypic expression,^1^ such as early-onset atrioventricular (AV) block, supraventricular and ventricular arrhythmia, progressive systolic heart failure, and frequent need for heart transplantation.^2^ LMNA cardiomyopathy has been associated with high incidence of malignant ventricular arrhythmia (MVA). In 25% of patients with LMNA cardiomyopathy, sudden cardiac death was the first presentation of MVA.^3^ MVA often occurs before the development of DCM.^2^ Sustained monomorphic ventricular tachycardia (VT) is seen in 35% of patients within 7 years of diagnosis.^4^ Ventricular arrhythmia in LMNA cardiomyopathy is invariably related to a basal septal intramural substrate.^4^ After VT ablation, there is a high rate of refractory ventricular arrhythmia (91% in 7 months) in patients with LMNA cardiomyopathy owing to the deep septal substrate.^4^

We present a case of a patient with LMNA cardiomyopathy who had a significant reduction in ventricular arrhythmia after receiving bilateral stellate ganglionectomy (also known as thoracic sympathectomy). There are no prior case reports that detail the clinical utility of this procedure for ventricular arrhythmia reduction in patients with LMNA cardiomyopathy. This case presents a novel therapy for management of refractory ventricular arrhythmia in this patient population.

## Case report

**Figure 1:**
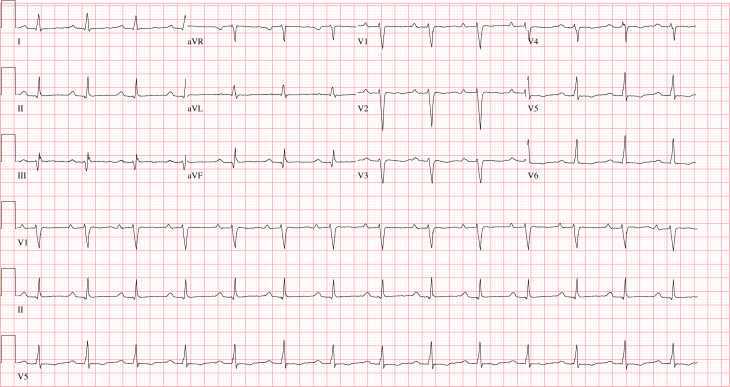
Baseline electrocardiogram after initial presentation demonstrating sinus rhythm with occasional premature ventricular contractions and first-degree atrioventricular block with evidence of prolonged QTc >500.

**Figure 2:**
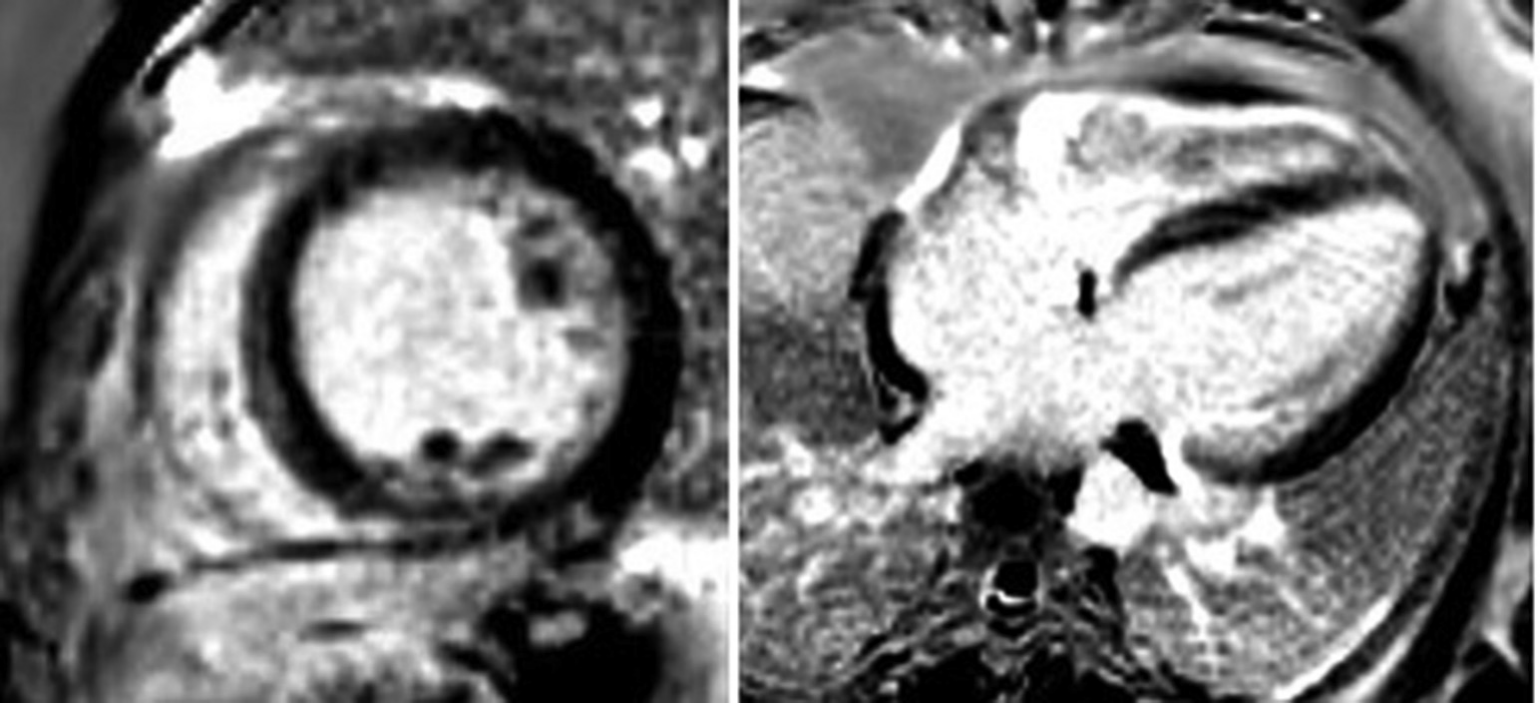
Cardiac magnetic resonance imaging with short axis at the level of the papillary muscle (*left*) and long axis 4-chamber (*right*) demonstrating no evidence of late gadolinium enhancement.

A 41-year-old man with no significant past medical history, who was a competitive mixed martial arts athlete, had his index presentation to our cardiac intensive care unit after suffering an out-of-hospital ventricular fibrillation (VF) arrest while playing with his dog at home. He was successfully resuscitated in the field with cardiopulmonary resuscitation and defibrillation. During the course of his hospitalization, he was found to have nonischemic DCM (left ventricular ejection fraction [LVEF] of 15%). Admission electrocardiogram demonstrated sinus tachycardia and first-degree AV block with prolonged QTc >500 (Figure 1), which subsequently normalized 5 days after admission. A left heart catheterization demonstrated no angiographic evidence of coronary artery disease. Cardiac magnetic resonance imaging demonstrated DCM with an LVEF of 30%–35%. (Figure 2A and 2B). There was no evidence of late gadolinium enhancement. Intravenous epinephrine challenge using the protocol described by Vyas and colleagues^5^ was negative. Procainamide challenge was deferred owing to frequent premature ventricular complexes present on the day of the study. Further nonischemic cardiomyopathy work-up with serum TSH, HIV, ANA, and iron studies was unremarkable. The patient was subsequently discharged with a Medtronic dual-chamber implantable cardioverter-defibrillator (ICD). Notably, the patient reported a significant family history of sudden cardiac death affecting his mother and multiple family members on his mother’s side in the fourth decade of life. Genetic testing with the Invitae Arrhythmia and Cardiomyopathy Comprehensive Panel identified no pathogenic mutations but reported a heterozygous c.618C>G (p.Phe206Leu) variant of uncertain significance in the *LMNA* gene. At 46 months after index presentation Invitae reclassified the mutation as pathogenic for LMNA cardiomyopathy.

The patient was treated initially with amiodarone therapy that subsequently was withdrawn owing to overall stability. Thereafter he was maintained on a beta-blocker. Overall, he fared well until suffering an ICD shock for VF 28 months after index presentation. Device interrogation also showed low paroxysmal atrial fibrillation burden. Sotalol and apixaban were initiated, but the patient could not tolerate the former owing to excessive fatigue. He suffered another ICD shock for torsades de pointes 29 months after index presentation. Sotalol was discontinued and he was subsequently transitioned back to amiodarone. Between 30 and 33 months after index presentation, ventricular pacing increased from 56.7% to 99.1%. Owing to concerns for pacing-induced deterioration in left ventricular function, he underwent upgrade to biventricular ICD 33 months after index presentation. The patient had another VF episode resulting in ICD shock. Ablation of VF triggers was considered. However, review of near- and far-field ventricular electrograms recorded by the ICD demonstrated that premature ventricular contractions triggering VF were of different morphologies, suggesting that such an approach would be unsuccessful. On account of recurrent ICD shocks despite antiarrhythmic therapy, the patient underwent bilateral stellate ganglionectomy 35 months after index presentation. The procedure was performed via a video-assisted thoracoscopic approach. A double-lumen endotracheal tube was utilized for 1 lung ventilation to allow for sequential bilateral sympathectomy. The left side was approached first. With video-assisted thoracoscopic exposure the first, second, third, and fourth rib heads were identified. The sympathetic chain and stellate ganglion were identified prior to division from T2 to T4. At T3, accessory nerves of Kuntz were identified and divided as well. Amiodarone therapy was discontinued 36 months after index presentation. At the time of this manuscript preparation, 57 months after index presentation, the patient remains off antiarrhythmic medications, with only 2 episodes of monomorphic tachycardia that were successfully terminated with antitachycardia pacing and no episodes of VF or ICD shocks. He has frequent multifocal premature ventricular depolarizations. He is currently being treated with guideline-directed medical therapy for heart failure with reduced systolic function and has NYHA class I symptoms. We recommended exercise limitation.

## Discussion

The Lamin A/C gene is mapped to the long arm of chromosome 1 (1q21.2-q21.3) and encodes 2 isoforms by alternative splicing, Lamin A and C.^6^ Lamin proteins are a type V intermediate filament that are major components of the scaffolding system of the inner nuclear membrane.^6^ Lamin mutations result in defects in the myocardium, skeletal muscles, and cardiac conduction system.^6^ LMNA cardiomyopathy has been associated with various arrhythmias, out of proportion to other heritable causes of DCM. They can range from relatively benign arrhythmia such as first-degree AV block and atrial fibrillation to such malignant arrhythmia as VT and VF. The 2017 AHA/ACC/HRS guidelines designate a class IIa recommendation that ICD can be beneficial in patients with LMNA cardiomyopathy and 2 or more risk factors (nonsustained VT, LVEF <45%, non–missense mutation, and male sex).^7^

Vigorous exercise has been shown to worsen cardiac disease in arrhythmogenic cardiomyopathy, such as arrhythmogenic right ventricular cardiomyopathy (ARVC). A recent study by Skjølsvik and colleagues^8^ demonstrated that active LMNA cardiomyopathy patients had worse systolic function compared with sedentary LMNA cardiomyopathy patients. Additional studies are needed to determine if exercise restriction should be recommended to patients with LMNA cardiomyopathy.

Our case demonstrates the malignant arrhythmic phenotype with which patients with LMNA cardiomyopathy frequently present. Patients often present in youth and there is early progression to advanced-stage heart failure with recurrent ventricular arrhythmia refractory to medical therapy. The autonomic nervous system is essential to the initiation and maintenance of ventricular arrhythmia.^9^ Over half a century ago, cardiac sympathetic denervation was shown to increase the VF threshold.^10^ Cardiac sympathetic denervation has been shown to be a viable treatment option for certain types of congenital long QT syndrome and catecholaminergic polymorphic VT.^11^ More recently, cardiac sympathetic denervation has been demonstrated to have a role in managing refractory ventricular arrhythmia in arrhythmogenic cardiomyopathy such as ARVC.

Although there are reports of bilateral cardiac sympathetic denervation (BCSD) in patients with ARVC, to our knowledge this is the first report of BCSD in a patient with LMNA cardiomyopathy. There are phenotypic similarities between LMNA cardiomyopathy and ARVC, as discussed earlier, which suggest shared arrhythmic substrate and mechanisms. Additionally, there are reports that describe genetic overlap between LMNA cardiomyopathy and ARVC.^12^^,^^13^ Assis and colleagues^14^ demonstrated the success of BCSD in patients with ARVC. After BCSD, 63% of patients with ARVC had no recurrent ventricular arrhythmia within approximately 2 years of follow-up. This report demonstrates the clinical utility of BCSD in patients with other forms of arrhythmogenic cardiomyopathy.^15^^,^^16^ Its utility in LMNA cardiomyopathy has not previously been reported. Our patient’s predominant arrhythmias were VF, for which sympathetic modulation may have greater efficacy than that seen for scar-related reentrant VT. Future studies are needed to establish BCSD as a treatment option for patients with refractory ventricular arrhythmia from LMNA cardiomyopathy.

**Key Teaching Points**

• Lamin A/C (LMNA) cardiomyopathy is a familial dilated cardiomyopathy with a high incidence of malignant ventricular arrhythmia and possible need for cardiac transplantation.
• Ventricular arrhythmia can present before the onset of heart failure.
• Stellate ganglionectomy (thoracic sympathectomy) is a treatment option for malignant arrhythmia associated with LMNA cardiomyopathy.
